# Supplementary material for: Modified rice bran arabinoxylan as a nutraceutical in health and disease—A scoping review with bibliometric analysis
Source: PLoS One. 2023 Aug 31;18(8):e0290314. doi: 10.1371/journal.pone.0290314 (PMC10470915; doi:10.1371/journal.pone.0290314)
Supplement: S1 File — (PDF) [file pone.0290314.s001.pdf]

S1. Supporting Materials for Methods

S1.1 Search strategy example

Name of the information source and the platform/service provider used

MEDLINE (via PubMed)

All the search terms to be used (both keywords/text words and index terms should be included) and how they are to be combined using Boolean logic; the use of truncation and wildcards.

((Modified) OR (Hydroly*) OR (Fermented) OR (Bioprocessed)) AND (Rice Bran) AND ((arabinoxylan) OR (hemicellulose) OR (polysaccharide) OR (Exo-biopolymer))

OR

((Biobran) OR (MGN-3) OR (Ribraxx) OR (BRM4) OR (RBEP))

Planned limits (date, language, etc.)

MEDLINE - No limit

Search Conducted On

27 September 2022

Number of Records

207

Detail Query

(("modifiable"[All Fields] OR "modified"[All Fields] OR "modifier"[All Fields] OR "modifiers"[All Fields] OR "modifies"[All Fields] OR "modify"[All Fields] OR "modifying"[All Fields] OR "hydroly*"[All Fields] OR ("ferment"[All Fields] OR "fermentabilities"[All Fields] OR "fermentability"[All Fields] OR "fermentable"[All Fields] OR "fermentate"[All Fields] OR "fermentated"[All Fields] OR "fermentates"[All Fields] OR "fermentation"[MeSH Terms] OR "fermentation"[All Fields] OR "fermentations"[All Fields] OR "fermentative"[All Fields] OR "fermentatively"[All Fields] OR "fermentator"[All Fields] OR "fermented"[All Fields] OR "fermenter"[All Fields] OR "fermenters"[All Fields] OR "fermenting"[All Fields] OR "fermention"[All Fields] OR "ferments"[All Fields]) OR "Bioprocessed"[All Fields]) AND (("oryza"[MeSH Terms] OR "oryza"[All Fields] OR "rice"[All Fields]) AND "Bran"[All Fields]) AND ("arabinoxylan"[Supplementary Concept] OR "arabinoxylan"[All Fields] OR "arabinoxylans"[All Fields] OR ("hemicellulose"[Supplementary Concept] OR "hemicellulose"[All Fields] OR "hemicelluloses"[All Fields] OR "hemicellulosic"[All Fields]) OR ("polysaccharid"[All Fields] OR "polysaccharide s"[All Fields] OR "polysaccharides"[MeSH Terms] OR "polysaccharides"[All Fields] OR "polysaccharide"[All Fields] OR "polysaccharidic"[All Fields] OR "polysaccharids"[All Fields]) OR "Exo-biopolymer"[All Fields])) OR ("polysaccharide mgn3"[Supplementary Concept] OR

"polysaccharide mgn3"[All Fields] OR "biobran"[All Fields] OR ("polysaccharide mgn3"[Supplementary Concept] OR "polysaccharide mgn3"[All Fields] OR "mgn 3"[All Fields]) OR "Ribraxx"[All Fields] OR "BRM4"[All Fields] OR "RBEP"[All Fields])

Translations

Modified: "modifiable"[All Fields] OR "modified"[All Fields] OR "modifier"[All Fields] OR "modifiers"[All Fields] OR "modifies"[All Fields] OR "modify"[All Fields] OR "modifying"[All Fields]

Fermented: "ferment"[All Fields] OR "fermentabilities"[All Fields] OR "fermentability"[All Fields] OR "fermentable"[All Fields] OR "fermentate"[All Fields] OR "fermentated"[All Fields] OR "fermentates"[All Fields] OR "fermentation"[MeSH Terms] OR "fermentation"[All Fields] OR "fermentations"[All Fields] OR "fermentative"[All Fields] OR "fermentatively"[All Fields] OR "fermentator"[All Fields] OR "fermented"[All Fields] OR "fermenter"[All Fields] OR "fermenters"[All Fields] OR "fermenting"[All Fields] OR "fermention"[All Fields] OR "ferments"[All Fields]

Rice: "oryza"[MeSH Terms] OR "oryza"[All Fields] OR "rice"[All Fields]

arabinoxylan: "arabinoxylan"[Supplementary Concept] OR "arabinoxylan"[All Fields] OR "arabinoxylans"[All Fields]

hemicellulose: "hemicellulose"[Supplementary Concept] OR "hemicellulose"[All Fields] OR "hemicelluloses"[All Fields] OR "hemicellulosic"[All Fields]

polysaccharide: “polysaccharid” [All Fields] OR “polysaccharide's"[All Fields] OR "polysaccharides"[MeSH Terms] OR "polysaccharides"[All Fields] OR "polysaccharide"[All Fields] OR "polysaccharidic"[All Fields] OR "polysaccharids"[All Fields]

Biobran: "polysaccharide MGN3"[Supplementary Concept] OR "polysaccharide MGN3"[All Fields] OR "biobran"[All Fields]

MGN-3: "polysaccharide MGN3"[Supplementary Concept] OR "polysaccharide MGN3"[All Fields] OR "mgn 3"[All Fields]

S 1.2. Data extraction instrument

Citation details (APA 7th)	
Country	
Article Type	Full paper, short communication, abstract only, thesis, book chapter, study protocol, trial registration.
Study Type	Human / Animal / In vitro / Others<br><br>*Human: Randomized controlled trials, non-randomized controlled trials, before and after studies and interrupted time-series studies, prospective or retrospective cohort studies, case-control studies, analytical cross-sectional studies, case series, individual case reports and descriptive cross-sectional studies.*<br><br>*Trial registration (Human trial only)*
Context	*Human: Population, sex, age, sample size, health/disease conditions.*<br>*Animal: Type, sex, age, sample size, health/disease conditions.*<br>*In vitro: Cell lines*
Methodology / methods	
Intervention & comparator	*If applicable, include duration, dosage, mode of administration.*
Outcome measures	
Key findings that relate to the scoping review question	

S1.3. Co-author weight coefficient scheme

The following formula is used to attribute the weight coefficient c(k,n), where n = number of authors, k= author order:

$c(1, n) = c(n,n) = c(\text{corresponding author}, n) = 1$.

$c(2,3) = 0.7$.

For $n \geqslant 4$, $2 \leqslant k \leqslant n - 1$:

$c(k,n) = 2(n - k + 1)/(n + 1)(n - 2)$.
