# Supplementary material for: Modified rice bran arabinoxylan as a nutraceutical in health and disease—A scoping review with bibliometric analysis
Source: PLoS One. 2023 Aug 31;18(8):e0290314. doi: 10.1371/journal.pone.0290314 (PMC10470915; doi:10.1371/journal.pone.0290314)
Supplement: S3 File — (PDF) [file pone.0290314.s003.pdf]

S3. Data Tables

1. Article

This table contains data for each included article.

#	Data Item	Format	Description
1	UID	Integer	Unique article identifier in database
2	Article ID	Author (Year)	Unique article identifier in following the APA 7th in-text citation Author (Year) format.
3	No. Author	Integer	Number of authors
4	Year	Year (YYYY)	Year of publication
5	Title	Text	Full article title
6	Published In	Text	The name of the publication (journal/book/conference, etc.)
7	Country	Text	Country of origin. If the authors were from more that one country, the country of origin is where the study or experiment was conducted.
8	Language	Text	Language of publication.
9	Google Scholar Citation	Integer	The citation count reported by Google Scholar
9	Article Type	Selection	Full paper, Short Communication, Book Chapter, Abstract, Thesis, Study Protocol or Trial Registration.
10	Is Human Study	Yes/No	Indicate whether it is a human study.
11	Is Animal Study	Yes/No	Indicate whether it is an animal study.
12	Is Cell Study	Yes/No	Indicate whether it is an in vitro cell study.
13	Is Chemical Analysis	Yes/No	Indicate whether it is a chemical analysis study.
14	RBAC Type	Text	The name of RBAC used in the study.
15	RBAC Source	Text	The commercial source of RBAC.
Data items for human study only			
16	Study Protocol Only	Yes/No	Is this article a study protocol?
17	Clinical Study Type	Text	Specify the type of clinical study design, e.g., RCT, non-RCT, before and after study, etc., if relevant.
18	Case Report Type	Selection	Individual case report or case series, if relevant.
19	Registered Trial?	Yes/No	Is this a registered trial?
20	Trial Registry	Text	Name of the trial registry, if registered.
21	Trial Registration no.	Text	The unique trial registration number, if registered.

2. Author

This table contains data for every author of the included article.

#	Data Item	Format	Description
1	Author ID	Text	Unique author ID in database
2	Full Name	Text	Full name of the author
3	Affiliation	Text	Author's affiliating institution. If more than one affiliation is reported, only the primary / latest affiliation is entered.
4	Country	Text	The country where the author conducted the work.

3. Authorship

This table specifies the author and article relationship.

#	Data Item	Format	Description
1	Article ID	Author (Year)	Unique article identifier in following the APA 7th in-text citation Author (Year) format.
2	Author ID	Text	Unique author ID in database
3	Author Affiliation	Text	Author's affiliating institution. If more than one affiliation is reported, on entry is recorded for each affiliated institution.
4	Affiliation Country	Text	The country of the affiliated institution
5	Is Corresponding?	Yes / No	Is this the corresponding author for the article?
6	Author Order	Sequential	A sequential number indicating the author's order in the author list starting from 1.
7	Weight Coefficient	Numeral	See Supplementary S4.

4. Institution

This table contains data for every author-affiliation of the included article.

#	Data Item	Format	Description
1	Name	Text	Name of an institution
2	Country	Text	The country where this institution is located
3	Type	Selection	The institution type being one of Academic, Healthcare, Private Practice, Research Institution, or Commercial Company.

5. Reference

This table captures the internal referencing across the included articles.

#	Data Item	Format	Description
1	Source Article ID	Author (Year)	The source article.
2	Reference Article ID	Author (Year)	The article being referenced/cited.

6. MeSH

#	Data Item	Format	Description
1	Article ID	Author (Year)	The source article.
2	MeSHTerms (OnDemand)	Text	MeSH terms generated from MeSH on Demand
3	MeSHTerms (PubMed)	Text	MeSH terms from PubMed, if available.

7. Condition-Action-Outcome

This table captures health or disease conditions investigated in each article, the potential beneficial actions, and the positive outcomes reported.

#	Data Item	Format	Description
1	Article ID	Author (Year)	The source article.
2	Study Design	Selection	Type of study design which reported the results.
2	Co-Intervention	Text	List of co-intervention with RBAC, e.g., chemotherapy, radiotherapy, etc.
3	Condition	Text	Name of the health conditions or disease.
4	Primary Site	Text	Primary body site or organ affected by the condition or investigated by the study.
5	Secondary Site	Text	Secondary body site or organ affected by the condition or investigated by the study.
6	Beneficial Action	Text	Beneficial action reported.
7	Outcome	Text	List of positive outcomes.
