# Supplementary material for: Modified rice bran arabinoxylan as a nutraceutical in health and disease—A scoping review with bibliometric analysis
Source: PLoS One. 2023 Aug 31;18(8):e0290314. doi: 10.1371/journal.pone.0290314 (PMC10470915; doi:10.1371/journal.pone.0290314)
Supplement: S4 File — (PDF) [file pone.0290314.s004.pdf]

S4. Additional tables and figures

Table S4.1. The characteristics of all included articles in alphabetic order of the first author's name (excluding trial registrations).

Article ID	Country	Language	Type	RBAC Type	Source	Design	T#	Condition	Action / Effect
Ali et al. (2012)	USA	English	Full paper	Biobran MGN-3	Daiwa	RCT	T1	Healthy	Immunomodulation
An (2011)	South Korea	Korean	Thesis	RBEP/EFR	Erom	Animal	T0	Cancer	Anticancer
						Animal	T0	Allergy	Antiallergy
						Cell	T0	Healthy	Noncytotoxic
						Chemical	T0	Healthy	Antioxidant
Badr El-Din et al. (2008)	Egypt	English	Full paper	Biobran MGN-3	Daiwa	Animal	T0	Cancer	Anticancer
Badr El-Din et al. (2016a)	Egypt	English	Full paper	Biobran MGN-3	Daiwa	Animal	T0	Cancer	Chemoprevention
Badr El-Din et al. (2016b)	Egypt	English	Full paper	Biobran MGN-3	Daiwa	Animal	T0	Cancer	Synergistic anticancer effect
Badr El-Din et al. (2016c)	Egypt	English	Full paper	Biobran MGN-3	Daiwa	Animal	T0	Cancer	Chemoprevention
									Hepatoprotection
Badr El-Din et al. (2019)	Egypt	English	Full paper	Biobran MGN-3	Daiwa	Animal	T0	Cancer	Synergistic anticancer effect
Badr El-Din et al. (2020)	Egypt	English	Full paper	Biobran MGN-3	Daiwa	Animal	T0	Cancer	Chemoprevention
									Hepatoprotection
Bae et al. (2004)	South Korea	Korean	Full paper	Biobran MGN-3	Daiwa	Animal	T0	Healthy	Immunomodulation
						Animal	T0	Cancer	Anticancer
						Animal	T0	Allergy	Antiallergy
Bang et al. (2010)	Vietnam	English	Full paper	Biobran MGN-3	Daiwa	RCT	T2	Cancer	Synergistic anticancer effect
Brush et al. (2010)	USA	English	Abstract	Biobran MGN-3	Daiwa	Cell	T0	Cancer	Anticancer
Cadden et al. (2020)	USA	English	Abstract	Biobran MGN-3	Daiwa	RCT	T1	HIV	No significant effect
Chae et al. (2004)	South Korea	Korean	Full paper	Biobran MGN-3	Daiwa	Cell	T0	Healthy	Immunomodulation
Choi et al. (2014)	South Korea	English	Full paper	RBEP/EFR	Erom	RCT	T1	Healthy	Immunomodulation
Cholujova et al. (2009)	Slovakia	English	Full paper	Biobran MGN-3	Daiwa	Cell	T0	Healthy	Immunomodulation
Cholujova et al. (2013)	Slovakia	English	Full paper	Biobran MGN-3	Daiwa	RCT	T1	Cancer	Immunomodulation

Chung et al. (2015)	South Korea	English	Full paper	BPP/FF	STR Biotech	Cell	T0	Healthy	Hepatoprotection
						Animal	T0	Hepatitis / Liver Disease	Hepatoprotection
Daizo et al. (2001)	Japan	Japanese	Abstract	Biobran MGN-3	Daiwa	Animal	T0	Hepatitis / Liver Disease	Hepatoprotection
Egashira et al. (2013)	Japan	English	Abstract	Biobran MGN-3	Daiwa	Animal	T0	Hepatitis / Liver Disease	Hepatoprotection
Elsaid et al. (2018)	Egypt	English	Full paper	Biobran MGN-3	Daiwa	RCT	T1	Geriatric	Immunomodulation
Elsaid et al. (2020)	Egypt	English	Full paper	Biobran MGN-3	Daiwa	RCT	T1	Geriatric	Psychoneuroimmuno-modulation
Elsaid et al. (2021)	Egypt	English	Full paper	Biobran MGN-3	Daiwa	RCT	T2	Cold / Flu	Antiflu
								Geriatric	Antiflu
Endo and Kanbayashi (2003)	Canada	English	Short Comm	Biobran MGN-3	Daiwa	Animal	T0	Healthy	Antiwasting
Ghoneum and Abedi (2004)	USA	English	Full paper	Biobran MGN-3	Daiwa	Animal	T0	Geriatric	Immunomodulation
						Cell	T0	Geriatric	Immunomodulation
Ghoneum and Agrawal (2011)	USA	English	Full paper	Biobran MGN-3	Daiwa	Cell	T0	Healthy	Immunomodulation
Ghoneum and Agrawal (2014)	USA	English	Full paper	Biobran MGN-3	Daiwa	Cell	T0	Healthy	Immunomodulation
Ghoneum and Brown (1999)	USA	English	Chapter	Biobran MGN-3	Daiwa	Bef-Aft	T1	Cancer	Immunomodulation
Ghoneum and El Sayed (2021)	USA	English	Full paper	Biobran MGN-3	Daiwa	Animal	T0	Alzheimer's disease	Memory enhancer
									Antioxidant
Ghoneum and Gollapudi (2003)	USA	English	Full paper	Biobran MGN-3	Daiwa	Cell	T0	Cancer	Anticancer
Ghoneum and Gollapudi (2005a)	USA	English	Full paper	Biobran MGN-3	Daiwa	Cell	T0	Cancer	Synergistic anticancer effect
Ghoneum and Gollapudi (2005b)	USA	English	Full paper	Biobran MGN-3	Daiwa	Cell	T0	Cancer	Synergistic anticancer effect
Ghoneum and Gollapudi (2011)	USA	English	Full paper	Biobran MGN-3	Daiwa	Cell	T0	Cancer	Synergistic anticancer effect
Ghoneum and Jewett (2000)	USA	English	Full paper	Biobran MGN-3	Daiwa	Cell	T0	Healthy	Immunomodulation
Ghoneum and Matsuura (2004)	USA	English	Full paper	Biobran MGN-3	Daiwa	Cell	T0	Healthy	Immunomodulation
Ghoneum (1998a)	USA	English	Full paper	Biobran MGN-3	Daiwa	Cell	T1	HIV	Antiretroviral
Ghoneum (1998b)	USA	English	Full paper	Biobran MGN-3	Daiwa	Bef-Aft	T0	Healthy	Immunomodulation
Ghoneum (1999)	USA	English	Abstract	Biobran MGN-3	Daiwa	Bef-Aft	T1	Cancer	Immunomodulation
								Chemical exposure	Immunomodulation
Ghoneum et al. (2000)	USA	English	Abstract	Biobran MGN-3	Daiwa	Cell	T0	Cancer	Anticancer

Ghoneum et al. (2008)	USA	English	Full paper	Biobran MGN-3	Daiwa	Cell	T0	Healthy	Antibacterial
Ghoneum et al. (2013)	USA	English	Full paper	Biobran MGN-3	Daiwa	Animal	T0	Healthy	Radioprotection<br>Antioxidant
Ghoneum et al. (2014)	USA	English	Full paper	Biobran MGN-3	Daiwa	Cell	T0	Cancer	Synergistic anticancer effect
Giese et al. (2008)	USA	English	Full paper	Biobran MGN-3	Daiwa	Animal	T0	Healthy	Immunomodulation
Gollapudi and Ghoneum (2008)	USA	English	Full paper	Biobran MGN-3	Daiwa	Cell	T0	Cancer	Synergistic anticancer effect
Golombick et al. (2016)	Australia	English	Full paper	Biobran MGN-3	Daiwa	Bef-Aft	T1	Cancer	Immunomodulation
Hajtó and Kirsch (2013)	Hungary	English	Full paper	Biobran MGN-3	Daiwa	Case Rep	T1	Cancer	Synergistic anticancer effect
Hajtó (2017)	Hungary	English	Full paper	Biobran MGN-3	Daiwa	Case Series	T1	Cancer	Synergistic anticancer effect
Hajtó (2018)	Hungary	English	Full paper	Biobran MGN-3	Daiwa	Case Series	T1	Cancer	Synergistic anticancer effect
Hajtó et al. (2015)	Hungary	English	Full paper	Biobran MGN-3	Daiwa	Case Series	T1	Cancer	Synergistic anticancer effect
Hajtó et al. (2016a)	Hungary	English	Full paper	Biobran MGN-3	Daiwa	Case Series	T1	Cancer	Synergistic anticancer effect
Hajtó et al. (2016b)	Hungary	English	Full paper	Biobran MGN-3	Daiwa	Cross-Sect	T1	Cancer	Psychoneuroimmuno-modulation
Hoshino et al. (2010)	Japan	English	Full paper	Biobran MGN-3	Daiwa	Cell	T0	Healthy	Antiinflammation
Ichihashi (2004)	Japan	Japanese	Full paper	Biobran MGN-3	Daiwa	Case Rep	T1	Rheumatism	Antirheumatic effect<br>Antiinflammation
Itoh et al. (2015)	Japan	English	Full paper	Biobran MGN-3	Daiwa	RCT	T1	Cancer<br>Gastroenteritis	Immunomodulation<br>Gastroprotection
Jacoby et al. (2001)	USA	English	Full paper	Biobran MGN-3	Daiwa	Animal	T0	Healthy	Chemoprotection<br>Gastroprotection
Kaketani (2004)	Japan	Japanese	Full paper	Biobran MGN-3	Daiwa	Case Series	T1	Cancer	Synergistic anticancer effect
Kambayashi and Endo (2002)	Canada	Japanese	Abstract	Biobran MGN-3	Daiwa	Animal	T0	Allergy	Antiasthma<br>Antiinflammation
Kamiya et al. (2014)	Japan	English	Full paper	Biobran MGN-3	Daiwa	RCT	T1	Irritable bowel syndrome	Immunomodulation
Kang et al. (2022)	South Korea	Korean	Full paper	RBEP/EFR	Erom	Cell	T0	Healthy	Immunomodulation
Kawai (2004)	Japan	Japanese	Full paper	Biobran MGN-3	Daiwa	Case Series	T1	Cancer	Synergistic anticancer effect

Kenyon (2001)	UK	English	Full paper	Biobran MGN-3	Daiwa	Bef-Aft	T1	Chronic fatigue syndrome	Antifatigue
Kim D.J. et al. (2011a)	S.Korea	Korean	Full paper	Fermented SuperC3GHi bran	Erom	Animal	T0	Cancer	Anticancer
						Animal	T0	Allergy	Antiinflammation
						Cell	T0	Healthy	Antiallergy
Kim D.J. et al. (2011b)	South Korea	Korean	Full paper	Fermented black rice bran	STR Biotech	Animal	T0	Healthy	Immunomodulation
Kim H.Y. et al. (2005)	South Korea	English	Full paper	RBEP/EFR	Erom	Animal	T0	Healthy	Immunomodulation
	South Korea					Cell	T0	Healthy	Immunomodulation
Kim H.Y. et al. (2007)	South Korea	English	Full paper	RBEP/EFR	Erom	Animal	T0	Cancer	Anticancer
Kim J.M. et al. (2020)	South Korea	English	Full paper	ONS	Erom	NRCT	T1	Cancer	Psychoneuroimmuno-modulation
Kim S.P. et al. (2013)	South Korea	English	Full paper	BPP/FF	STR Biotech	Cell	T0	Healthy	Immunomodulation
	South Korea					Animal	T0	Endotoxemia	Antioxidant
	South Korea					Animal	T0	Hepatitis / Liver Disease	Hepatoprotection
Kim S.P. et al. (2014)	South Korea	English	Full paper	BPP/FF	STR Biotech	Cell	T0	Healthy	Immunomodulation
	South Korea					Animal	T0	Bacterial infection	Antibacterial
Kim S.P. et al. (2018)	South Korea	English	Full paper	BPRBE	STR Biotech	Cell	T0	Healthy	Immunomodulation
						Animal	T0	Gastroenteritis	Antibacterial
Lewis et al. (2020a)	USA	English	Full paper	Biobran MGN-3	Daiwa	RCT	T1	HIV	Endothelial improvement
Lewis et al. (2020b)	USA	English	Full paper	Biobran MGN-3	Daiwa	RCT	T1	HIV	Immunomodulation
Lewis et al. (2020c)	USA	English	Full paper	Biobran MGN-3	Daiwa	RCT	T1	Hepatitis / Liver Disease	Hepatoprotection
Lissoni et al. (2008)	Italy	English	Full paper	Biobran MGN-3	Daiwa	Bef-Aft	T1	Cancer	Immunomodulation
Markus et al. (2006)	USA	English	Full paper	Biobran MGN-3	Daiwa	Case Series	T1	Cancer	Anticancer
Masood et al. (2013)	Pakistan	English	Full paper	Biobran MGN-3	Daiwa	RCT	T1	Cancer	Psychoneuroimmuno-modulation
McDermott et al. (2006)	UK	English	Full paper	Biobran MGN-3	Daiwa	RCT	T1	Chronic fatigue syndrome	No significant effect
Meshitsuka (2013)	Japan	English	Full paper	Biobran MGN-3	Daiwa	Case Series	T1	Cancer	Synergistic anticancer effect
Miura et al. (2004/2013)	Japan	English	Chapter	Biobran MGN-3	Daiwa	Cell	T0	Healthy	Immunomodulation
Noaman et al. (2008)	Egypt	English	Full paper	Biobran MGN-3	Daiwa	Animal	T0	Cancer	Anticancer

									Antioxidant
Ohara et al. (2000)	Japan	English	Full paper	Biobran MGN-3	Daiwa	Animal	T0	Diabetes mellitus	Antihyperlipidemic effect
									Taste influencer
Ohara et al. (2002)	Japan	English	Full paper	Biobran MGN-3	Daiwa	Animal	T0	Diabetes mellitus	Antihyperglycemic effect
									Antihyperlipidemic effect
Okamura (2004)	Japan	Japanese	Full paper	Biobran MGN-3	Daiwa	Case Rep	T1	Cancer	Synergistic anticancer effect
									Immunomodulation
									Hepatoprotection
Ooi et al. (2020)	Australia	English	Protocol	Biobran MGN-3	Daiwa	NA	T1	NA	NA
Pérez-Martínez et al. (2015)	Spain	English	Full paper	Biobran MGN-3	Daiwa	Cell	T0	Healthy	Immunomodulation
						Animal	T0	Cancer	Immunomodulation
Pescatore et al. (2022)	USA	English	Full paper	Biobran MGN-3	Daiwa	Bef-Aft	T1	Cancer	Antimetastatic effect
Petrovics et al. (2016)	Hungary	English	Full paper	Biobran MGN-3	Daiwa	RCT	T1	Chronic fatigue syndrome	Antifatigue
								Cancer	Psychoneuroimmuno-modulation
Salama et al. (2016)	Egypt	English	Full paper	Biobran MGN-3	Daiwa	RCT	T1	Hepatitis / Liver Disease	Hepatoprotection
									Antiviral
Sudo et al. (2001)	Japan	Japanese	Full paper	Biobran MGN-3	Daiwa	Animal	T0	Endotoxemia	Antiinflammation
Takahara and Sano (2004)	Japan	Japanese	Full paper	Biobran MGN-3	Daiwa	RCT	T2	Cancer	Psychoneuroimmuno-modulation
Tan and Flores (2020)	Philippines	English	Full paper	Biobran MGN-3	Daiwa	RCT	T2	Cancer	Synergistic anticancer effect
									Radioprotection
									Psychoneuroimmuno-modulation
Tazawa et al. (2000)	Japan	English	Full paper	Biobran MGN-3	Daiwa	Chemical	T0	Oxidative stress	Antioxidant
Tazawa et al. (2003)	Japan	Japanese	Full paper	Biobran MGN-3	Daiwa	RCT	T1	Cold / Flu	Antiflu
								Geriatric	Immunomodulation
Tsunekawa (2004)	Japan	Japanese	Full paper	Biobran MGN-3	Daiwa	Case Rep	T1	Cancer	Synergistic anticancer effect
									Psychoneuroimmuno-modulation
Yamada et al. (2002)	Japan	Japanese	Abstract	Biobran MGN-3	Daiwa	Animal	T0	Hepatitis / Liver Disease	Hepatoprotection

Yu et al. (2004)	South Korea	English	Full paper	RBEP/EFR	Erom	Cell	T0	Healthy	Immunomodulation
Zhao et al. (2020)	China	English	Full paper	Biobran MGN-3	Daiwa	Animal	T0	Gastroenteritis	Radioprotection<br>Antiinflammation<br>Antioxidant
Zheng et al. (2012a)	Japan	English	Full paper	Biobran MGN-3	Daiwa	Animal	T0	Hepatitis / Liver Disease	Hepatoprotection<br>Antiinflammation
Zheng et al. (2012b)	Japan	English	Full paper	Biobran MGN-3	Daiwa	Animal	T0	Hepatitis / Liver Disease	Hepatoprotection<br>Antiinflammation
Zhu et al. (2017)	Japan	English	Full paper	Biobran MGN-3	Daiwa	Cell	T0	Healthy	Antiangiogenesis

Abbreviations: Bef-Aft, before and after study**;** BPP/FF, bioprocessed polysaccharide or fermented black rice bran; BPRBE, bioprocessed rice bran extract; Cross-Sect**,** descriptive cross-sectional study**;** NA, not available**;** NRCT**,** non-randomised controlled trial**;** ONS, oral nutritional supplement; RBEP/EFR**,** rice bran bio-exopolymer or Erom's fermented rice bran**;** Rep, report**;** RCT, randomised controlled trial; T#, translational research spectrum classification (T1 – T4); UK, United Kingdom; USA, United States of America.

Table S4.2. The research output by country according to article count and authors' total weighted coefficient (TWC).

Ranking By Article	Ranking By Author	Ranking By TWC	Country	No. Authors	No. Institutions	Publishing Period	Count by Article's Country (% Total) [1]	Count by Author's Country (% Total) [2]	Author's TWC
1	1	1	USA	65	19	1988 - 2022	29 (29.59%)	43 (43.88%)	78.04
2	2	2	Japan	80	23	2000 - 2017	22 (22.45%)	27 (27.55%)	56.71
3	3	3	South Korea	44	18	2004 - 2022	15 (15.31%)	15 (15.31%)	42.16
4	4	4	Egypt	16	5	2008 - 2021	11 (11.22%)	14 (14.29%)	22.54
5	5	5	Hungary	12	1	2013 - 2018	7 (7.14%)	7 (7.14%)	14.37
6	7	6	Australia	12	7	2016 - 2020	2 (2.04%)	2 (2.04%)	6.77
6	7	9	Canada	2	1	2002 - 2003	2 (2.04%)	2 (2.04%)	4.00
6	6	8	UK	7	6	2001 - 2015	2 (2.04%)	3 (3.06%)	4.05
6	7	7	Slovakia	8	2	2009 - 2013	2 (2.04%)	2 (2.04%)	5.70
7	7	10	China	5	4	2015 - 2020	1 (1.02%)	2 (2.04%)	3.00
7	8	12	Italy	11	4	2008 - 2008	1 (1.02%)	1 (1.02%)	2.00
7	8	11	Pakistan	3	1	2013 - 2013	1 (1.02%)	1 (1.02%)	2.70
7	8	12	Philippines	2	1	2020 - 2020	1 (1.02%)	1 (1.02%)	2.00
7	8	15	Spain	8	4	2015 - 2015	1 (1.02%)	1 (1.02%)	1.73
7	8	14	Vietnam	8	1	2010 - 2010	1 (1.02%)	1 (1.02%)	1.90
7	8	17	Germany	4	1	2015 - 2015	1 (1.02%)	1 (1.02%)	1.22
-	8	18	Iraq	1	1	2019 - 2019	0 (0.00%)	1 (1.02%)	0.40
-	7	16	Switzerland	1	1	2013 - 2015	0 (0.00%)	2 (2.04%)	1.33

Note: [1.] An article's country of origin is based on where the study is being conducted or the first author's country.

[2.] The count by author's country is based on co-authorship, with all co-authors' countries counting the same article as research output.

Table S4.3. Funding statement from all included articles.

ArticleID	Funding Statement
Ali et al. (2012)	This study was supported by a gift from Daiwa Pharmaceutical Inc., Tokyo, Japan, which is the manufacturer of RBAC and research and training funds from the Laboratory for Clinical and Biological Studies at the University of Miami, Miller School of Medicine. Daiwa Pharmaceutical Inc. did not participate in the design of this study.
An (2011)	Not disclosed
Badr El-Din et al. (2008)	We thank Daiwa Pharmaceutical Co. LTD, Tokyo, Japan, for the financial support of this project.
Badr El-Din et al. (2016a)	This work was supported by Diawa Pharmaceutical Co Ltd, Tokyo, Japan; Grant #T0099108 (M. Ghoneum); and by NIH-NIMHD Grants U54MD007598 (formerly U54RR026138) and S21 MD000103 (D. Pan).
Badr El-Din et al. (2016b)	The authors would like to acknowledge Daiwa Pharmaceutical Co. Ltd., Tokyo, Japan, for the financial support of this project, Grant #T0099108.
Badr El-Din et al. (2016c)	Not disclosed
Badr El-Din et al. (2019)	Not disclosed
Badr El-Din et al. (2020)	Partial support was provided by Daiwa Pharmaceutical Co., Ltd., Tokyo, Japan.
Bae et al. (2004)	This study was carried out by the support of the research fund of the Institute of Life Science of EromLife.
Bang et al. (2010)	Not disclosed
Brush et al. (2010)	Not disclosed
Cadden et al. (2020)	Not disclosed
Chae et al. (2004)	This study was supported by research funds from the Erom Life Institute of Life Sciences.
Choi et al. (2014)	This work was supported by “Food Functionality Evaluation program” under the Ministry of Agriculture, Food and Rural Affairs, and partly Korea Food Research Institute and Erom.
Cholujova et al. (2009)	This work was supported by a commercial grant from Daiwa Pharmaceutical Co. Ltd.
Cholujova et al. (2013)	The study was supported by research funding from Daiwa Pharmaceutical to J.S.
Chung et al. (2015)	This study was supported by grants from the Republic of Korea’s Small and Medium Business Administration (SMBA) (S1061057).
Daizo et al. (2001)	Not disclosed
Egashira et al. (2013)	Not disclosed

Elsaid et al. (2018)	The authors would like to acknowledge Daiwa Pharmaceutical Co., Ltd., Tokyo, Japan, for supplying Biobran/MGN-3 for this study. This study was supported in part by NIH-NIMHD grant nos. U54MD007598 and NIH/NCATS grant nos. UL1TR000124 and S21 MD000103.
Elsaid et al. (2020)	The authors would like to thank Daiwa Pharmaceutical Co., Ltd., Tokyo, Japan for providing Biobran/MGN-3.
Elsaid et al. (2021)	This research received no external funding. The authors gratefully acknowledge Daiwa Pharmaceutical Co., Ltd. for providing Biobran/MGN-3.
Endo & Kanbayashi (2003)	Not disclosed
Ghoneum & Abedi (2004)	Not disclosed
Ghoneum & Agrawal (2011)	Not disclosed
Ghoneum & Agrawal (2014)	This work was supported by grant #C0030300 from Daiwa Pharmaceutical Co., Ltd., Japan.
Ghoneum & Brown (1999)	Not disclosed
Ghoneum & El Sayed (2021)	Biobran/MGN-3 was provided by Daiwa Pharm. Co., Ltd, Japan. This work was funded by Diawa Pharmaceutical Co., Ltd., Tokyo, Japan; Grant #T0099108.
Ghoneum & Gollapudi (2003)	This work was supported by a grant from Daiwa Pharmaceutical Company, Tokyo, Japan.
Ghoneum & Gollapudi (2005a)	The authors would also like to thank Daiwa Pharmaceutical Co. LTD, Tokyo, Japan for the financial support of this project.
Ghoneum & Gollapudi (2005b)	The authors would also like to thank Daiwa Pharmaceutical Co., Tokyo, Japan, Ltd., for the financial support of this project.
Ghoneum & Gollapudi (2011)	Not disclosed
Ghoneum & Jewett (2000)	Not disclosed
Ghoneum & Matsuura (2004)	This work was supported by grant #D22988 from Daiwa Pharmaceutical Co., LTD. Tokyo, Japan.
Ghoneum (1998b)	Not disclosed
Ghoneum (1998a)	Not disclosed
Ghoneum (1999)	Not disclosed
Ghoneum et al. (2000)	MGN-3, commercially known as Bio Bran, was provided by Daiwa Pharmaceuticals Company. Ltd., Tokyo, Japan. Suported in part by VA Medical Research Funds and by funds provided by Daiwa Pharmaceuticals Company, Ltd.
Ghoneum et al. (2013)	This work was supported by Diawa Pharmaceutical Co. Ltd, Tokyo, Japan; Grant #T0099108.
Ghoneum et al. (2014)	The Authors would like to acknowledge Daiwa Pharmaceutical Co. Ltd., Tokyo, Japan, for the financial support of this project.

Ghoneum et al. (2008)	This work was supported by grant #D22988 from Daiwa Pharmaceutical Co. Ltd., Tokyo, Japan.
Giese et al. (2008)	Not disclosed
Gollapudi & Ghoneum (2008)	The authors would like to acknowledge Daiwa Pharmaceutical Co. Ltd., Tokyo, Japan, for the financial support of this project.
Golombick et al. (2016)	Terry Golombick received partial funding support during the study period. The other authors received no financial support. The publication costs are borne by Biomedica.
Hajtó & Kirsch (2013)	Not disclosed
Hajtó (2017)	Not disclosed
Hajtó (2018)	Not disclosed
Hajtó et al. (2015)	The authors thank Mrs. Cornelia van den Bergh (for her grants to support this research).
Hajtó et al. (2016a)	The authors thank Mrs. Cornelia van den Bergh (for her grants to support this research).
Hajtó et al. (2016b)	Not disclosed
Hoshino et al. (2010)	Not disclosed
Ichihashi (2004)	Not disclosed
Itoh et al. (2015)	The authors deeply appreciate Daiwa Pharmaceutical Co., Ltd., the manufacturer of both the HRB and placebo foods, which were provided free of charge.
Jacoby et al. (2001)	Not disclosed
Kaketani (2004)	Not disclosed
Kambayashi & Endo (2002)	Not disclosed
Kamiya et al. (2014)	They also thank Daiwa Pharmaceutical Co. Ltd. for supplying the powder of both Biobran and placebo and for the assistance of this paper submission. This study was supported, in part, by a grant of Japanese Society of Psychosomatic Medicine on Digestive Disease.
Kang et al. (2022)	Not disclosed
Kawai (2004)	Not disclosed
Kenyon (2001)	Not disclosed
Kim D.J. et al. (2011b)	Not disclosed
Kim D.J. et al. (2011a)	This study was funded by the Institute of Technology Planning and Evaluation (IPET) for Food, Agriculture, Forestry and Fisheries (Project No.: 108096-03-1-HD110).
Kim H.Y. et al. (2005)	This work was supported by the fund of Erom Corporation
Kim H.Y. et al. (2007)	Not disclosed

Kim J.M. et al.(2020)	This study was supported by High Value-added Food Technology Development Program, Ministry of Agriculture, Food and Rural Affairs, Republic of Korea (312006-3) and by the Medical Research Center Program (No. 2011-0030074) through National Research Foundation (NRF) grant funded by the Korean government (MSIP).
Kim S.P. et al. (2013)	Not disclosed
Kim S.P. et al. (2014)	Not disclosed
Kim S.P. et al. (2018)	This work was supported by the Korean Institute of Planning and Evaluation for Technology in Food, Agricultural, Forestry and Fisheries (IPET) through Agri-Bio Industry Technology Development Program, funded by Ministry of Agriculture, Food and Rural Affairs (MAFRA) (no. 314019–3), and by the Korean Institute of Planning and Evaluation for Technology in Food, Agricultural, Forestry and Fisheries (IPET) through High Value-added Food Technology Development Program, funded by Ministry of Agriculture, Food and Rural Affairs (MAFRA) (no. 314076–3).
Lewis et al. (2020b)	This work was supported by a gift from Daiwa Health Development. The study was also supported by Grant Number 1UL1TR000460, Miami Clinical and Translational Science Institute, from the National Center for Advancing Translational Sciences and the National Institute on Minority Health and Health Disparities.
Lewis et al. (2020a)	This work was supported by a gift from Daiwa Health Development. The study was also supported by Grant Number 1UL1TR000460, Miami Clinical and Translational Science Institute, from the National Center for Advancing Translational Sciences and the National Institute on Minority Health and Health Disparities.
Lewis et al. (2020c)	This work was supported by a gift from Daiwa Health Development. The study was also supported by (Grant no. 1UL1TR000460) Miami Clinical and Translational Science Institute from the National Center for Advancing Translational Sciences and the National Institute on Minority Health and Health Disparities.
Lissoni et al. (2008)	Not disclosed
Markus et al. (2006)	Not disclosed
Masood et al. (2013)	Not disclosed
McDermott et al. (2006)	The trial was funded by Daiwa Pharmaceutical Company. Dr George Lewith's post is funded by a grant from the Rufford Maurice Laing Foundation.
Meshitsuka (2013)	Not disclosed
Miura et al. (2004/2013)	Not disclosed
Noaman et al. (2008)	We thank Daiwa Pharmaceuticals, Co. Ltd, Tokyo, Japan, for generously providing the MGN-3.
Ohara et al. (2000)	This work was supported in part by Grant-in-Aid for scientific research from Kobe Women's University
Ohara et al. (2002)	Not disclosed
Okamura (2004)	Not disclosed

Ooi et al. (2020)	Daiwa Pharmaceutical Co., Ltd. (Daiwa) provided financial funding to commence the RBAC-QoL project with the af filiating university of the corresponding author as the trial sponsor. BioMedica Nutraceuti cals Pty Ltd (BioMedica) provided additional funding to conduct the gut microbiome study of this research. The RBAC and placebo powder will be manufactured and supplied by Daiwa. SLO is a recipient of the Australian Government Research Training Program scholarship for this study.
Pérez-Martínez et al. (2015)	We thank Daiwa Pharmaceutical Co. Ltd., Tokyo, Japan, for providing us MGN-3/Biobran. This work was supported in part by the National Health Service of Spain grant FIS PI12/01622, Fundación de la Sociedad Española de HematoOncologia Infantil and CRIS Cancer Foundation (http://www.criscancer.org/en/index.php) to Antonio Perez-Martínez and from the German Jose Carreras Leukemia Foundation and the German Childhood Cancer Foundation (DKS) to Matthias Pfeiffer.
Pescatore et al. (2022)	Not disclosed
Petrovics et al. (2016)	The Authors would like to acknowledge Daiwa Pharmaceutical Co. Ltd., Tokyo, Japan, for the technical material support of this project. (free samples of BioBran MGN-3). The manufacturers of BioBran funded the trial, but placed no restrictions on the design of the trial or on the publication of results.
Salama et al. (2016)	Funding for the study and Biobran were provided by Daiwa Pharmaceutical Co., Ltd., Tokyo, Japan. This study was supported in part by NIH-NIMHD grant no. U54 MD007598 and NIH-NCATS grant no. UL1 TR000124.
Sudo et al. (2001)	Not disclosed
Takahara & Sano (2004)	We would like thank Daiwa Pharmaceutical for supplying MGN-3 (Biobran) and Mitsubishi Chemical Laboratory (B.C.L.) for cooperation in blood test.
Tan & Flores (2020)	Research funding and Lentin and placebo sachets were provided by Daiwa Pharmaceuticals Co Ltd. Tokyo, Japan.
Tazawa et al. (2000)	Not disclosed
Tazawa et al. (2003)	Not disclosed
Tsunekawa (2004)	Not disclosed
Yamada et al. (2002)	Not disclosed
Yu et al. (2004)	This work was supported by the fund of Eromlife Corporation.
Zhao et al. (2020)	This work was supported by the Li Jie-shou Gut Barrier Foundation, China (Project nos. LJS-201708 and LJS-201811C), the Wuxi Science and Technology Bureau, China (Project no.201827), the Wuxi Health and Family Planning Commission Youth Research Foundation, China
Zheng et al. (2012a)	Not disclosed
Zheng et al. (2012b)	Not disclosed
Zhu et al. (2017)	Not disclosed

Table S4.4. Characteristics of trial registration included for assessment.

Trial Citation	Registry	Year	Registration ID	Condition	Product	Status	Research Output
ClinicalTrials.gov (2009)	ClinicalTrials.gov	2009	NCT01018381	Liver cancer<br>Hep B & C	Biobran MGN-3	Completed	Bang et al. (2010)
University Hospital Medical Information Network Center (2010)	UMIN-CTR (Japan)	2010	UMIN000004350	Gastroenteritis for cervical cancer	Biobran MGN-3	Completed	Itoh et al. (2015)
Clinical Research Information Service (2012)	CRiS (S. Korea)	2012	KCT0000536	Healthy	RBEP	Completed	Choi et al. (2014)
ClinicalTrials.gov (2013)	ClinicalTrials.gov	2013	NCT02019628	Healthy	Biobran MGN-3	Completed	Ali et al. (2012)
ClinicalTrials.gov (2014)	ClinicalTrials.gov	2014	NCT02214173	HIV	Biobran MGN-3	Completed	Lewis et al. (2020a); Lewis et al. (2020b)
ClinicalTrials.gov (2015)	ClinicalTrials.gov	2015	NCT02568787	Non-alcoholic liver disease	Biobran MGN-3	Completed	Lewis et al. (2020c)
ClinicalTrials.gov (2016a)	ClinicalTrials.gov	2016	NCT02690103	Hepatitis C infection	Biobran MGN-3	Completed	Salama et al. (2016)
ClinicalTrials.gov (2016b)	ClinicalTrials.gov	2016	NCT02922907	HIV	Biobran MGN-3	Completed	Cadden et al. (2020)
Current Controlled Trials (2017)	ISRCTN (UK)	2017	ISRCTN31318565	Lyme borreliosis	Biobran MGN-3	Ongoing	
Clinical Research Information Service (2018)	CRiS (S. Korea)	2018	KCT0002646	Healthy	STR RB-F	Discontinued	Trial interrupted by COVID-19 – no results.
University Hospital Medical Information Network Center (2018)	UMIN-CTR (Japan)	2018	UMIN000034569	B-cell non-Hodgkin's lymphoma	Biobran MGN-3	Completed	Results pending
Australian New Zealand Clinical Trials Registry (2019)	ANZCTR	2019	ACTRN12619000562178	Solid organ cancer	Biobran MGN-3	Ongoing	Ooi et al. (2020)
ClinicalTrials.gov (2020)	ClinicalTrials.gov	2020	NCT04646980	Influenza-like illnesses	Biobran MGN-3	Completed	Elsaid et al. (2021)

Table S4.5. Top 10 authors ranked by the total weighted coefficient (TWC) and article count.

Ranking By		Author	Affiliation	Country	Count (% Total)	TWC
TWC	Count					
1	1	Ghoneum, Mamdooh	Charles Drew University of Medicine and Science	USA	30 (30.61%)	30.00
2	2	Badr El-Din, Nariman K.	University of Mansoura	Egypt	9 (9.18%)	7.64
3	4	Gollapudi, Sastry	University of California at Irvine	USA	6 (6.12%)	6.00
3	4	Hajtó, Tibor	University of Pécs	Hungary	6 (6.12%)	6.00
5	3	Maeda, Hiroaki	Daiwa Pharmaceutical Co. Ltd.	Japan	7 (7.14%)	5.36
6	4	Egashira, Yukari	Chiba University	Japan	6 (6.12%)	4.50
7	10	Lewis, John E.	University of Miami Miller School of Medicine	USA	4 (4.08%)	4.00
8	4	Hong, Seong Gil	Erom Co. Ltd.	South Korea	6 (6.12%)	3.75
9	-	Elsaid, Ahmed F.	Zagazig University	Egypt	3 (3.06%)	3.00
9	-	Friedman, Mendel	U.S. Department of Agriculture	USA	3 (3.06%)	3.00
9	-	Kim, Sung Phil	Ajou University	South Korea	3 (3.06%)	3.00
9	-	Konefal, Janet	University of Miami Miller School of Medicine	USA	3 (3.06%)	3.00
9	-	Nam, Seok Hyun	Ajou University	South Korea	3 (3.06%)	3.00
-	8	Hwang, Sung Joo	Erom Co. Ltd.	South Korea	5 (5.10%)	1.51
-	8	Lee, Seong Ae	STR Biotech Co. Ltd.	South Korea	5 (5.10%)	1.66
-	10	Ali, Doaa A.	University of Mansoura	Egypt	4 (4.08%)	2.14
-	10	Kim, Hwa Young	Erom Co. Ltd.	South Korea	4 (4.08%)	2.46
-	10	Kim, Jin Min	Erom Co. Ltd.	South Korea	4 (4.08%)	0.79
-	10	Park, Mi Hyoun	Erom Co. Ltd.	South Korea	4 (4.08%)	2.31
	10	Shaheen, Magda	Charles Drew University of Medicine and Science	USA	4 (4.08%)	1.42
-	10	Woolger, Judi M.	University of Miami Miller School of Medicine	USA	4 (4.08%)	0.27

Table S4.6. Cancer sites investigated by the included articles ordered by article count and breakdown by study design.

#	Cancer Site	Count (%)	Preclinical (%)	Observational (%)	Interventional (%)
1	Various	9 (20%)	-	2 (22.22%)	7 (77.78%)
2	Breast	8 (17.78%)	4 (50%)	3 (37.5%)	1 (12.5%)
3	Liver	5 (11.11%)	2 (40%)	-	2 (50%)
4	Blood *	4 (8.89%)	2 (50%)	-	2 (50%)
5	Colorectal	2 (4.44%)	-	2 (100%)	-
6	Lung	2 (4.44%)	-	2 (100%)	-
7	Ovary	2 (4.44%)	-	2 (100%)	-
8	Stomach	2 (4.44%)	1 (50%)	1 (50%)	-
9	Skin	2 (4.44%)	1 (50%)	1 (50%)	-
10	Cervical	1 (2.22%)	-	-	1 (100%)
11	Head & Neck	1 (2.22%)	-	-	1 (100%)
12	Bile Duct	1 (2.22%)	-	1 (100%)	-
13	Pancreatic	1 (2.22%)	-	1 (100%)	-
14	Umbilical	1 (2.22%)	-	1 (100%)	-
15	Uterus	1 (2.22%)	-	1 (100%)	-
16	Prostate	1 (2.22%)	1 (100%)	-	-

Note: * cancer of the blood includes multiple myeloma, leukemia, and early B-cell lymphoid malignancies.

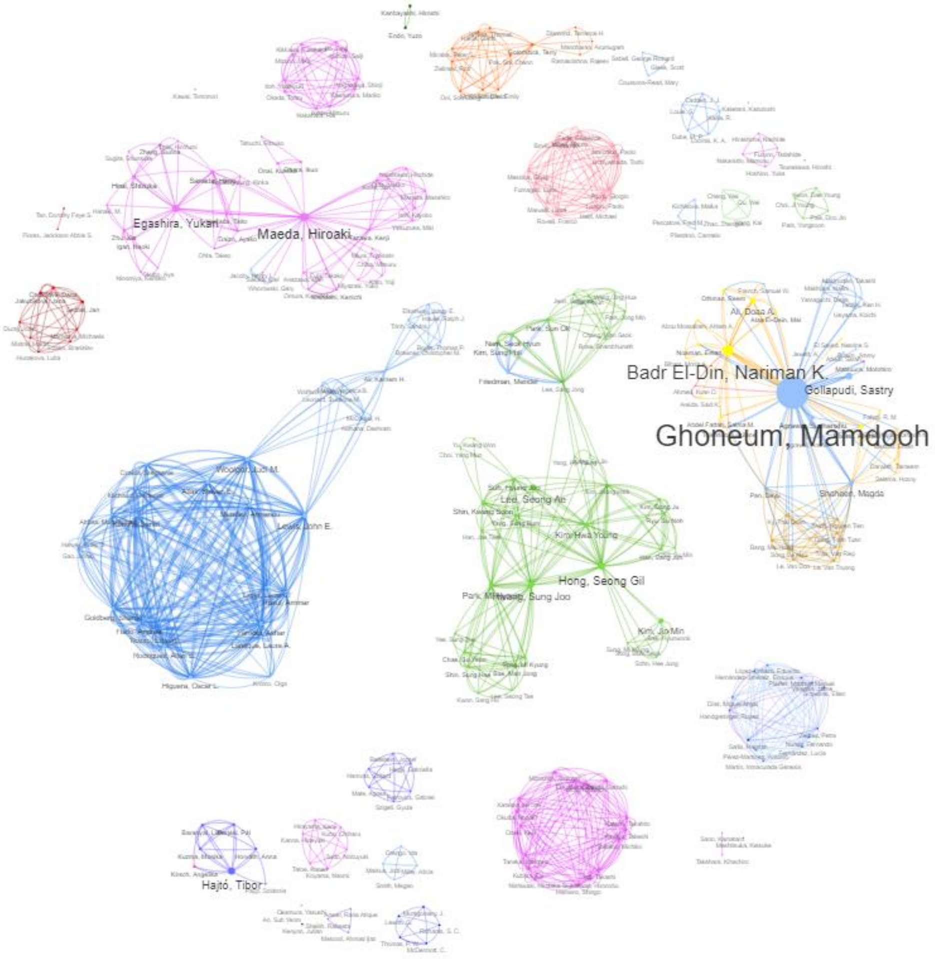

Fig. S4.1. The collaborative network of authors in RBAC research. The interactive version is available online at http://resource.rbac-gol.info.

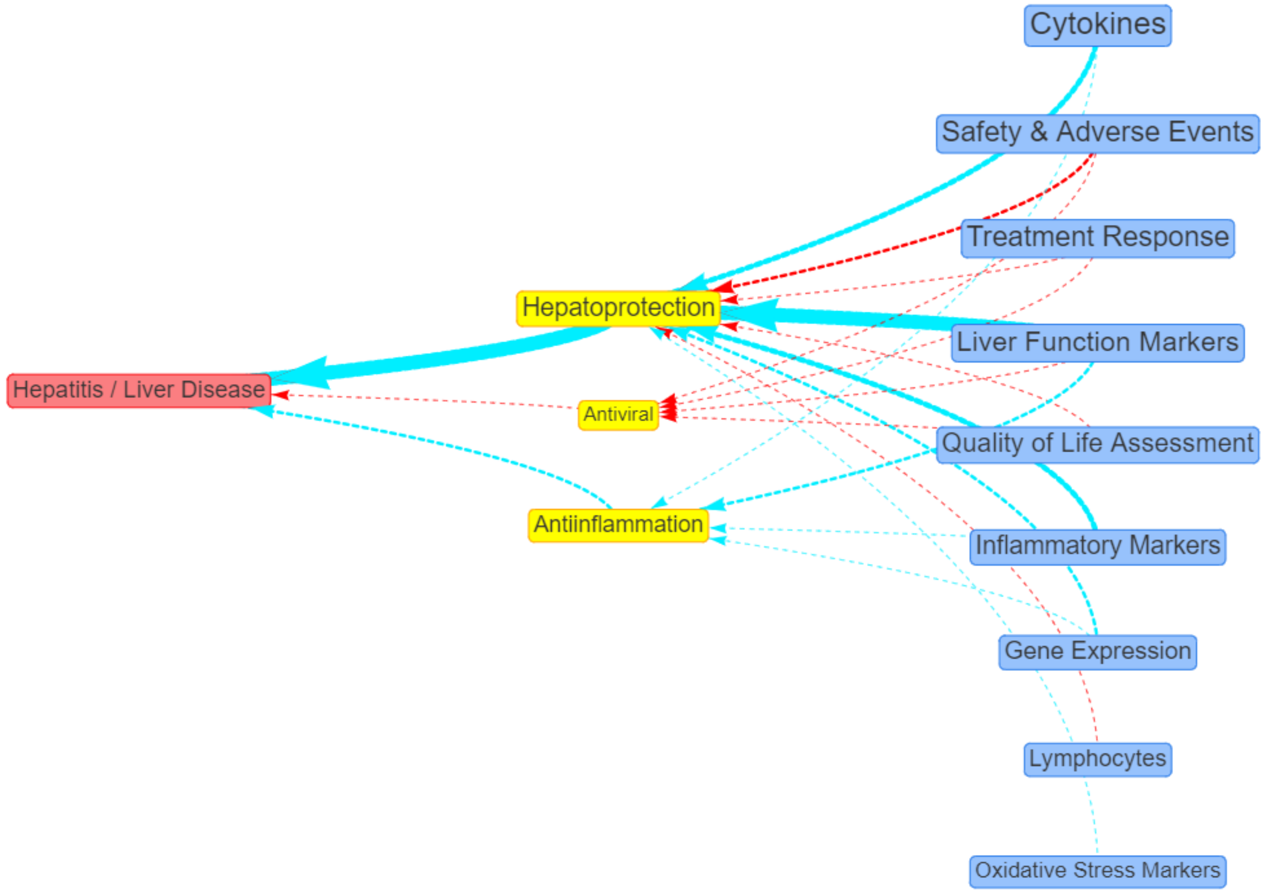

Fig. S4.2. Network visualisation of the beneficial actions (yellow nodes) of RBAC in hepatitis and liver diseases (red node) and its associated positive outcomes (blue). The links between nodes represent the availability of sources of evidence, with the colours indicating the study types (red = interventional, green = observational, and blue = preclinical). The node size and link thickness reflect the number of sources. The original interactive network diagram is available at https://resource.rbac-qol.info
