# Supplementary material for: Modified rice bran arabinoxylan as a nutraceutical in health and disease—A scoping review with bibliometric analysis
Source: PLoS One. 2023 Aug 31;18(8):e0290314. doi: 10.1371/journal.pone.0290314 (PMC10470915; doi:10.1371/journal.pone.0290314)
Supplement: S5 File — (PDF) [file pone.0290314.s005.pdf]

S5. Additional References

Full citation of all included articles and trial registrations in APA 7th format

Ali, K. H., Melillo, A. B., Leonard, S. M., Asthana, D., Woolger, J. M., Wolfson, A. H., McDaniel, H., & Lewis, J. E. (2012). An open-label, randomized clinical trial to assess the immunomodulatory activity of a novel oligosaccharide compound in healthy adults. *Functional Foods in Health and Disease, 2*(7), 265.

An, S. Y. (2011). *Immune-enhance and anti-tumor effect of exo-biopolymer extract from submerged culture of Lentinus edodes with rice bran* [Doctoral Thesis, Korea University]. Seoul, Republic of Korea.

Australian New Zealand Clinical Trials Registry. (2019). *Rice bran arabinoxylan compound and quality of life of cancer patients (RBAC-QoL)*. Australian New Zealand Clinical Trials Registry. Retrieved 2022-12-01 from https://www.anzctr.org.au/Trial/Registration/TrialReview.aspx?id=377340&isReview=true

Badr El-Din, N. K., Abdel Fattah, S. M., Pan, D., Tolentino, L., & Ghoneum, M. (2016a). Chemopreventive activity of MGN-3/Biobran against chemical induction of glandular stomach carcinogenesis in rats and its apoptotic effect in gastric cancer cells. *Integrative Cancer Therapies, 15*(4), NP26-NP34. https://doi.org/10.1177/1534735416642287

Badr El-Din, N. K., Ali, D. A., Alaa El-Dein, M., & Ghoneum, M. (2016b). Enhancing the apoptotic effect of a low dose of paclitaxel on tumor cells in mice by arabinoxylan rice bran (MGN-3/Biobran). *Nutrition and Cancer, 68*(6), 1010-1020. https://doi.org/10.1080/01635581.2016.1192204

Badr El-Din, N. K., Ali, D. A., & Othman, R. M. (2016c). Inhibition of experimental carcinogenesis by the bioactive natural product Biobran. *Journal of Plant Protection and Pathology, 7*(1), 85-91. https://doi.org/10.21608/jppp.2016.50064

Badr El-Din, N. K., Ali, D. A., Othman, R. M., French, S. W., & Ghoneum, M. (2020). Chemopreventive role of arabinoxylan rice bran, MGN-3/Biobran, on liver carcinogenesis in rats. *Biomedicine & Pharmacotherapy, 126*, 110064. https://doi.org/10.1016/j.biopha.2020.110064

Badr El-Din, N. K., Areida, S. K., Ahmed, K. O., & Ghoneum, M. (2019). Arabinoxylan rice bran (MGN-3/Biobran) enhances radiotherapy in animals bearing Ehrlich ascites carcinoma. *Journal of Radiation Research, 60*(6), 747-758. https://doi.org/10.1093/jrr/rrz055

Badr El-Din, N. K., Noaman, E., & Ghoneum, M. (2008). In vivo tumor inhibitory effects of nutritional rice bran supplement MGN-3/Biobran on Ehrlich carcinoma-bearing mice. *Nutrition and Cancer, 60*(2), 235-244. https://doi.org/10.1080/01635580701627285

Bae, M. J., Lee, S. T., Chae, S. Y., Shin, S. H., Kwon, S. H., Park, M. H., Song, M. Y., & Hwang, S. J. (2004). The effects of the arabinoxylane and the polysaccharide peptide (PSP) on the antiallergy, anticancer. *Journal of the Korean Society of Food Science and Nutrition, 33*(3), 469-474. https://doi.org/10.3746/jkfn.2004.33.3.469

Bang, M. H., Tran, V. R., Thinh, N. T., Song, L. H., Dung, T. T., Le, V. T., Le, V. D., Ky, T. D., Pan, D., Shaheen, M., & Ghoneum, M. (2010). Arabinoxylan rice bran (MGN-3) enhances the effects of interventional therapies for the treatment of hepatocellular carcinoma: a three-year randomized clinical trial. *Anticancer Research, 30*(12), 5145-5151.

Brush, T. P., Trinh, S., Brawner, C. M., Ali, K. H., Hauke, R. J., & Elkahwaji, J. E. (2010). RBAC, a modified form of Arabinoxylan from rice bran, impairs prostate cancer cell line proliferation, adhesion, and invasion in vitro [Abstract]. *Cancer Research, 70*(8), 5662-5662. https://doi.org/10.1158/1538-7445.AM10-5662

Cadden, J. J., Loomis, K. A., Kallia, R., Louie, S., & Dube, M. P. (2020). Anti-inflammatory effects of arabinoxylan rice bran supplementation in participants with treated, suppressed HIV infection and inadequate immune reconstitution: a randomized, doubleblind trial [Abstract]. *Antiviral Therapy, 25*(Suppl 1), A26.

Chae, S. Y., Shin, S. H., Bae, M. J., Park, M. H., Song, M. K., Hwang, S. J., & Yee, S. T. (2004). Effect of arabinoxylane and PSP on activation of immune cells. *Journal of the Korean Society of Food Science and Nutrition, 33*(2), 278-286. https://doi.org/10.3746/jkfn.2004.33.2.278

Choi, J. Y., Paik, D. J., Kwon, D. Y., & Park, Y. (2014). Dietary supplementation with rice bran fermented with Lentinus edodes increases interferon-γ activity without causing adverse effects: a randomized, double-blind, placebo-controlled, parallel-group study. *Nutrition Journal, 13*, 35. https://doi.org/10.1186/1475-2891-13-35

Cholujova, D., Jakubikova, J., Czako, B., Martisova, M., Hunakova, L., Duraj, J., Mistrik, M., & Sedlak, J. (2013). MGN-3 arabinoxylan rice bran modulates innate immunity in multiple myeloma patients. *Cancer Immunology, Immunotherapy, 62*(3), 437-445. https://doi.org/10.1007/s00262-012-1344-z

Cholujova, D., Jakubikova, J., & Sedlak, J. (2009). BioBran-augmented maturation of human monocyte-derived dendritic cells. *Neoplasma, 56*(2), 89-95. https://doi.org/10.4149/neo_2009_02_89

Chung, W. S., Wang, J. H., Bose, S., Park, J. M., Park, S. O., Lee, S. J., Jeon, S., & Kim, H. (2015). Hepatoprotective effect of Lentinus edodes mycelia fermented formulation against alcoholic liver injury in rats. *Journal of Food Biochemistry, 39*(3), 251-262. https://doi.org/10.1111/jfbc.12124

Clinical Research Information Service. (2012). *Effects of ingestion of fermented rice bran powder on immune function in healthy adults*. Clinical Research Information Service (CRiS) of Republic of Korea https://cris.nih.go.kr/cris/search/listDetail.do?searchWord=KCT0000536

Clinical Research Information Service. (2018). *A 8 week, randomized, double-blind, Placebo-controlled clinical trial of RB-F for the evaluation of efficacy and safety on immune function*. Clinical Research Information Service (CRiS) of Republic of Korea https://cris.nih.go.kr/cris/search/listDetail.do?searchWord=KCT0002646

ClinicalTrials.gov. (2009). *Arabinoxylan rice bran (MGN-3/Biobran) for the treatment of hepatocellular carcinoma and hepatitis B and C infection*. National Library of Medicine (US). https://clinicaltrials.gov/show/NCT01018381

ClinicalTrials.gov. (2013). *Enhanced natural killer cell activity and RBAC*. National Library of Medicine (US). https://clinicaltrials.gov/show/NCT02019628

ClinicalTrials.gov. (2014). *The effect of an enhanced rice bran nutritional supplement in HIV(BRM4).* National Library of Medicine (US). https://clinicaltrials.gov/ct2/show/NCT02214173

ClinicalTrials.gov. (2015). *The effect of an RBAC supplement (BRM4) on NAFLD*. National Library of Medicine (US). https://clinicaltrials.gov/show/NCT02568787

ClinicalTrials.gov. (2016a). *Response modifier (arabinoxylan rice bran/mgn-3/biobran) with interferon-alpha for for HCV*. National Library of Medicine (US). https://clinicaltrials.gov/show/NCT02690103

ClinicalTrials.gov. (2016b). *Rice bran supplementation in treated HIV infection*. National Library of Medicine (US). https://clinicaltrials.gov/show/NCT02922907

ClinicalTrials.gov. (2020). *Biobran/MGN-3 increases innate resistance and reduces the incidence of influenza-like illnesses*. National Library of Medicine (US). https://clinicaltrials.gov/show/NCT04646980

Current Controlled Trials. (2017). *Trial of the functional food Biobran in patients with persistent symptoms attributed to Lyme borreliosis*. BioMed Central. https://www.isrctn.com/ISRCTN31318565

Daizo, A., Yamada, T., Egashira, Y., Maeda, H., Ohta, T., & Sanada, H. (2001). Effect of enzymatically treated rice bran hemicellulose (MGN-3) on experimental galactosamine liver injury in rats [Abstract]. *Journal of Japanese Association for Dietary Fiber Research, 5*(2), 48-48. https://doi.org/10.11217/jjdf1997.5.2_43

Egashira, Y., Hanaki, M., Hirai, S., Zhu, X., & Igari, N. (2013). Suppressive effect of hydrolyzed rice bran (HRB) on D-galactosamine/ LPS induced IL-18 and hepatitis by inhibition of NF-kappa B pathway in mice [Abstract]. *Annals of Nutrition and Metabolism, 63*(Suppl 1), 1700-1701. https://doi.org/10.1159/000354245

Elsaid, A. F., Agrawal, S., Agrawal, A., & Ghoneum, M. (2021). Dietary supplementation with Biobran/MGN-3 increases innate resistance and reduces the incidence of influenza-like illnesses in elderly subjects: a randomized, double-blind, placebo-controlled pilot clinical trial. *Nutrients, 13*(11). https://doi.org/10.3390/nu13114133

Elsaid, A. F., Fahmi, R. M., Shaheen, M., & Ghoneum, M. (2020). The enhancing effects of Biobran/MGN-3, an arabinoxylan rice bran, on healthy old adults' health-related quality of life: a randomized, double-blind, placebo-controlled clinical trial. *Quality of Life Research, 29*(2), 357-367. https://doi.org/10.1007/s11136-019-02286-7

Elsaid, A. F., Shaheen, M., & Ghoneum, M. (2018). Biobran/MGN-3, an arabinoxylan rice bran, enhances NK cell activity in geriatric subjects: A randomized, double-blind, placebo-controlled clinical trial. *Experimental and Therapeutic Medicine, 15*(3), 2313-2320. https://doi.org/10.3892/etm.2018.5713

Endo, Y., & Kanbayashi, H. (2003). Modified rice bran beneficial for weight loss of mice as a major and acute adverse effect of cisplatin. *Pharmacology and Toxicology, 92*(6), 300-303. https://doi.org/10.1034/j.1600-0773.2003.920608.x

Ghoneum, M. (1998a). Anti-HIV activity in vitro of MGN-3, an activated arabinoxylane from rice bran. *Biochemical and Biophysical Research Communications, 243*(1), 25-29.

Ghoneum, M. (1998b). Enhancement of human natural killer cell activity by modified arabinoxylane from rice bran (MGN-3). *International Journal of Immunotherapy, 14*(2), 89-99.

Ghoneum, M. (1999, Dec 11-13). *Immunostimulation and cancer prevention* [Abstract]. 7th International Congress on Anti-Aging & Biomedical Technologies, Las Vegas, NV, USA.

Ghoneum, M., & Abedi, S. (2004). Enhancement of natural killer cell activity of aged mice by modified arabinoxylan rice bran (MGN-3/Biobran). *Journal of Pharmacy and Pharmacology, 56*(12), 1581-1588. https://doi.org/10.1211/0022357044922

Ghoneum, M., & Agrawal, S. (2011). Activation of human monocyte-derived dendritic cells in vitro by the biological response modifier arabinoxylan rice bran (MGN-3/Biobran). *International Journal of Immunopathology and Pharmacology, 24*(4), 941-948. https://doi.org/10.1177/039463201102400412

Ghoneum, M., & Agrawal, S. (2014). MGN-3/biobran enhances generation of cytotoxic CD8+ T cells via upregulation of DEC-205 expression on dendritic cells. *International Journal of Immunopathology and Pharmacology, 27*(4), 523-530. https://doi.org/10.1177/039463201402700408

Ghoneum, M., Badr El-Din, N. K., Abdel Fattah, S. M., & Tolentino, L. (2013). Arabinoxylan rice bran (MGN-3/Biobran) provides protection against whole-body gamma -irradiation in mice via

restoration of hematopoietic tissues. *Journal of Radiation Research, 54*(3), 419-429. https://doi.org/10.1093/jrr/rrs119

Ghoneum, M., Badr El-Din, N. K., Ali, D. A., & Alaa El-Dein, M. (2014). Modified arabinoxylan from rice bran, MGN-3/Biobran, sensitizes metastatic breast cancer cells to paclitaxel in vitro. *Anticancer Research, 34*(1A), 81-87.

Ghoneum, M., & Brown, J. (1999). NK Immunorestoration and cancer patients by MGN-3, a modified arabinoxylan rice bran (Study of 32 patients followed for up to 4 years). In R. M. Klatz & R. Goldman (Eds.), *Anti-aging Medical Therapeutics* (Vol. III, pp. 217-226). Health Quest Publications.

Ghoneum, M., & El Sayed, N. S. (2021). Protective effect of Biobran/MGN-3 against sporadic Alzheimer's disease mouse model: possible role of oxidative stress and apoptotic pathways. *Oxidative Medicine and Cellular Longevity, 2021*. https://doi.org/10.1155/2021/8845064

Ghoneum, M., & Gollapudi, S. (2003). Modified arabinoxylan rice bran (MGN-3/Biobran) sensitizes human T cell leukemia cells to death receptor (CD95)-induced apoptosis. *Cancer Letters, 201*(1), 41-49. https://doi.org/10.1016/s0304-3835(03)00458-0

Ghoneum, M., & Gollapudi, S. (2005a). Modified arabinoxylan rice bran (MGN-3/Biobran) enhances yeast-induced apoptosis in human breast cancer cells in vitro. *Anticancer Research, 25*(2a), 859-870.

Ghoneum, M., & Gollapudi, S. (2005b). Synergistic role of arabinoxylan rice bran (MGN-3/Biobran) in S. cerevisiae-induced apoptosis of monolayer breast cancer MCF-7 cells. *Anticancer Research: International Journal of Cancer Research and Treatment, 25*(6b), 4187-4196.

Ghoneum, M., & Gollapudi, S. (2011). Synergistic apoptotic effect of arabinoxylan rice bran (MGN-3/Biobran) and curcumin (turmeric) on human multiple myeloma cell line U266 in vitro. *Neoplasma, 58*(2), 118-123. https://doi.org/10.4149/neo_2011_02_118

Ghoneum, M., & Jewett, A. (2000). Production of tumor necrosis factor-alpha and interferon-gamma from human peripheral blood lymphocytes by MGN-3, a modified arabinoxylan from rice bran, and its synergy with interleukin-2 in vitro. *Cancer Detection and Prevention, 24*(4), 314-324.

Ghoneum, M., & Matsuura, M. (2004). Augmentation of macrophage phagocytosis by modified arabinoxylan rice bran (MGN-3/biobran). *International Journal of Immunopathology and Pharmacology, 17*(3), 283-292. https://doi.org/10.1177/039463200401700308

Ghoneum, M., Matsuura, M., & Gollapudi, S. (2008). Modified arabinoxylan rice bran (MGN-3/Biobran) enhances intracellular killing of microbes by human phagocytic cells in vitro. *International Journal of Immunopathology and Pharmacology, 21*(1), 87-95. https://doi.org/10.1177/039463200802100110

Ghoneum, M., Tachiki, K. H., Ueyama, K., Makinodan, T., Makhijani, N., & Yamaguchi, D. (2000, Dec 14-17). *Natural biological response modifier (MGN-3) shown to be effective against tumor cell growth* [Abstract]. 8th International Congress on Anti-Aging & Biomedical Technologies, Las Vegas, NV, USA.

Giese, S., Sabell, G. R., & Coussons-Read, M. (2008). Impact of ingestion of rice bran and shitake mushroom extract on lymphocyte function and cytokine production in healthy rats. *Journal of Dietary Supplements, 5*(1), 47-61. https://doi.org/10.1080/19390210802329196

Gollapudi, S., & Ghoneum, M. (2008). MGN-3/Biobran, modified arabinoxylan from rice bran, sensitizes human breast cancer cells to chemotherapeutic agent, daunorubicin. *Cancer Detection and Prevention, 32*(1), 1-6. https://doi.org/10.1016/j.cdp.2008.02.006

Golombick, T., Diamond, T. H., Manoharan, A., & Ramakrishna, R. (2016). Addition of rice bran arabinoxylan to curcumin therapy may be of benefit to patients with early-stage B-cell lymphoid malignancies (monoclonal gammopathy of undetermined significance, smoldering multiple myeloma, or stage 0/1 chronic lymphocytic leukemia). *Integrative Cancer Therapies, 15*(2), 183-189. https://doi.org/10.1177/1534735416635742

Hajtó, T. (2017). Can a standardized plant immunomodulator (rice bran arabinoxylan concentrate/MGN-3) increase the effects of MEK and BRAF inhibitors with clinical benefit? Case report of a patient with carcinoma in biliary duct. *Research and Review Insights, 1*(3), 1-4. https://doi.org/10.15761/rri.1000115

Hajtó, T. (2018). New perspectives to improve the MHC-I unrestricted immune mechanisms against malignant tumors. *Advances in Clinical and Translational Research, 2*(3), 100014.

Hajtó, T., Baranyai, L., Kirsch, A., Kuzma, M., & Perjési, P. (2015). Can a synergistic activation of pattern recognition receptors by plant immunomodulators enhance the effect of oncologic therapy? Case Report of a patient with uterus and ovary sarcoma. *Clinical Case Reports and Reviews, 1*(10), 235-238. https://doi.org/10.15761/CCRR.1000176

Hajtó, T., Horváth, A., Baranyai, L., Kuzma, M., & Perjési, P. (2016a). Can the EGFR inhibitors increase the immunomodulatory effects of standardized plant extracts (mistletoe lectin and arabonoxylan) with clinical benefit? Case report of a patient with lung adenocarcinoma. *Clinical Case Reports and Reviews, 2*(6), 456-459. https://doi.org/10.15761/CCRR.1000244

Hajtó, T., Horváth, A., & Papp, S. (2016b). Improvement of quality of life in tumor patients after an immunomodulatory treatment with standardized mistletoe lectin and arabinoxylan plant extracts. *International Journal of Neurorehabilitation, 3*(2), 2-4. https://doi.org/10.4172/2376-0281.1000205

Hajtó, T., & Kirsch, A. (2013). Case reports of cancer patients with hepatic metastases treated by standardized plant immunomodulatory preparations. *Journal of Cancer Research Updates, 2*(1), 1-9. https://doi.org/10.6000/1929-2279.2013.02.01.1

Hoshino, Y., Hirashima, N., Nakanishi, M., & Furuno, T. (2010). Inhibition of degranulation and cytokine production in bone marrow-derived mast cells by hydrolyzed rice bran. *Inflammation Research, 59*(8), 615-625. https://doi.org/10.1007/s00011-010-0173-9

Ichihashi, K. (2004). Experience with administration of BioBran in patients with chronic rheumatism. *Clinical Pharmacology and Therapy, 14*(4), 459-463.

Itoh, Y., Mizuno, M., Ikeda, M., Nakahara, R., Kubota, S., Ito, J., Okada, T., Kawamura, M., Kikkawa, F., & Naganawa, S. (2015). A randomized, double-blind pilot trial of hydrolyzed rice bran versus placebo for radioprotective effect on acute gastroenteritis secondary to chemoradiotherapy in patients with cervical cancer. *Evidence-based Complementary and Alternative Medicine*, 974390-974390. https://doi.org/10.1155/2015/974390

Jacoby, H. I., Wnorowski, G., Sakata, K., & Maeda, H. (2001). The effect of MGN-3 on cisplatin and doxorubicin induced toxicity in the rat. *Journal of Nutraceuticals, Functional & Medical Foods, 3*(4), 3-11. https://doi.org/10.1300/J133v03n04_02

Kaketani, K. (2004). A case where an immunomodulatory food was effective in conservative therapy for progressive terminal pancreatic cancer. *Clinical Pharmacology and Therapy, 14*(3), 273-279.

Kambayashi, H., & Endo, Y. (2002). Evaluation of the effects of asthma prevention and symptom reduction by enzymatically modified rice-bran foods in asthmatic model mice [Abstract] *Japanese Journal of Allergology, 51*(9/10), 957-957. https://doi.org/10.15036/arerugi.51.957_3

Kamiya, T., Shikano, M., Tanaka, M., Ozeki, K., Ebi, M., Katano, T., Hamano, S., Nishiwaki, H., Tsukamoto, H., Mizoshita, T., Mori, Y., Kubota, E., Tanida, S., Kataoka, H., Okuda, N., & Joh, T. (2014). Therapeutic effects of Biobran, modified arabinoxylan rice bran, in improving symptoms of diarrhea predominant or mixed type irritable bowel syndrome: a pilot, randomized controlled study. *Evidence-Based Complementary and Alternative Medicine, 2014*. https://doi.org/10.1155/2014/828137

Kang, S. J., Yang, H. Y., Lee, S. J., Kim, J. H., Hwang, S. J., & Hong, S. G. (2022). Immunostimulatory effect of rice bran fermented by lentinus edodes mycelia on mouse macrophages and splenocytes. *Journal of the Korean Society of Food Science and Nutrition, 51*(8), 743-750. https://doi.org/10.3746/jkfn.2022.51.8.743

Kawai, T. (2004). One case of a patient with umbilical metastasis of recurrent cancer (Sister Mary Joseph's Nodule, SMJN) who has survived for a long time under immunomodulatory supplement therapy. *Clinical Pharmacology and Therapy, 14*(3), 281-288.

Kenyon, J. (2001, Nov). A descriptive questionnaire-based study on the use of Biobran (MGN3), in chronic fatigue syndrome. *Townsend Letter for Doctors and Patients*, (220), 48-50.

Kim, D. J., Choi, S. M., Kim, H. Y., Kim, J. H., Ryu, S. N., Han, S. J., & Hong, S. G. (2011a). Evaluation of biological activities of fermented rice bran from novel black colored rice cultivar SuperC3GHi. *Korean Journal of Crop Science, 56*(4), 420-426.

Kim, D. J., Ryu, S.-N., Han, S. J., Kim, H. Y., Kim, J. H., & Hong, S. G. (2011b). In vivo immunological activity in fermentation with black rice bran. *The Korean Journal of Food And Nutrition, 24*(3), 273-281. https://doi.org/10.9799/KSFAN.2011.24.3.273

Kim, H. Y., Han, J. T., Hong, S. G., Yang, S. B., Hwang, S. J., Shin, K. S., Suh, H. J., & Park, M. H. (2005). Enhancement of immunological activity in exo-biopolymer from submerged culture of Lentinus edodes with rice bran. *Natural Product Sciences, 11*(3), 183-187.

Kim, H. Y., Kim, J. H., Yang, S. B., Hong, S. G., Lee, S. A., Hwang, S. J., Shin, K. S., Suh, H. J., & Park, M. H. (2007). A polysaccharide extracted from rice bran fermented with Lentinus edodes enhances natural killer cell activity and exhibits anticancer effects. *Journal of Medicinal Food, 10*(1), 25-31. https://doi.org/10.1089/jmf.2006.116

Kim, J. M., Hong, S. G., Song, B. S., Sohn, H. J., Baik, H., & Sung, M. K. (2020). Efficacy of cereal-based oral nutrition supplement on nutritional status, inflammatory cytokine secretion and quality of life in cancer patients under cancer therapy. *Journal of Cancer Prevention, 25*(1), 55-63. https://doi.org/10.15430/JCP.2020.25.1.55

Kim, S. P., Lee, S. J., Nam, S. H., & Friedman, M. (2018). The composition of a bioprocessed shiitake (Lentinus edodes) mushroom mycelia and rice bran formulation and its antimicrobial effects against Salmonella enterica subsp. enterica serovar Typhimurium strain SL1344 in macrophage cells and in mice. *BMC Complementary and Alternative Medicine, 18*(1). https://doi.org/10.1186/s12906-018-2365-8

Kim, S. P., Park, S. O., Lee, S. J., Nam, S. H., & Friedman, M. (2013). A polysaccharide isolated from the liquid culture of Lentinus edodes (Shiitake) mushroom mycelia containing black rice bran protects mice against a Salmonella lipopolysaccharide-induced endotoxemia. *Journal of Agricultural and Food Chemistry, 61*(46), 10987-10994. https://doi.org/10.1021/jf403173k

Kim, S. P., Park, S. O., Lee, S. J., Nam, S. H., & Friedman, M. (2014). A polysaccharide isolated from the liquid culture of Lentinus edodes (Shiitake) mushroom mycelia containing black rice bran protects mice against salmonellosis through upregulation of the Th1 immune reaction. *Journal of Agricultural and Food Chemistry, 62*(11), 2384-2391. https://doi.org/10.1021/jf405223q

Lewis, J. E., Atlas, S. E., Abbas, M. H., Rasul, A., Farooqi, A., Lantigua, L. A., Michaud, F., Goldberg, S., Lages, L. C., Gao, J., Higuera, O. L., Fiallo, A., Harvey, P. D., Tiozzo, E., Woolger, J. M., Ciraula, S., Mendez, A., Rodriguez, A. E., & Konefal, J. (2020a). Cardiovascular, endothelial function,

and immune markers in response to treatment with a polysaccharide in HIV(+) adults in a randomized, double-blind placebo-controlled trial. *Journal of Clinical and Translational Research, 5*(3), 140-147.

Lewis, J. E., Atlas, S. E., Abbas, M. H., Rasul, A., Farooqi, A., Lantigua, L. A., Michaud, F., Goldberg, S., Lages, L. C., Higuera, O. L., Fiallo, A., Tiozzo, E., Woolger, J. M., Ciraula, S., Mendez, A., Rodriguez, A. E., & Konefal, J. (2020b). The novel effects of a hydrolyzed polysaccharide dietary supplement on immune, hepatic, and renal function in adults with HIV in a randomized, double-blind, placebo-control trial. *Journal of Dietary Supplements, 17*(4), 429-441. https://doi.org/10.1080/19390211.2019.1619010

Lewis, J. E., Atlas, S. E., Higuera, O. L., Fiallo, A., Rasul, A., Farooqi, A., Kromo, O., Lantigua, L. A., Tiozzo, E., Woolger, J. M., Goldberg, S., Mendez, A., Rodriguez, A. E., & Konefal, J. (2020c). Corrigendum to "The Effect of a Hydrolyzed Polysaccharide Dietary Supplement on Biomarkers in Adults with Nonalcoholic Fatty Liver Disease". *Evidence-Based Complementary and Alternative Medicine, 2020*, 10. https://doi.org/10.1155/2020/9575878

Lissoni, P., Messina, G., Brivio, F., Fumagalli, L., Rovelli, F., Maruelli, L., Miceli, M., Marchiori, P., Porro, G., Held, M., Fede, G., & Uchiyamada, T. (2008). Modulation of the anticancer immunity by natural agents: inhibition of T regulatory lymphocyte generation by arabinoxylan in patients with locally limited or metastatic solid tumors. *Cancer Therapy, 6*(2), 1011-1016.

Markus, J., Miller, A., Smith, M., & Orengo, I. (2006). Metastatic hemangiopericytoma of the skin treated with wide local excision and MGN-3. *Dermatologic Surgery, 32*(1), 145-147. https://doi.org/10.1111/1524-4725.2006.32023

Masood, A. I., Sheikh, R., & Anwer, R. A. (2013). "BIOBRAN MGN-3"; Effect of reducing side effects of chemotherapy in breast cancer patients. *The Professional Medical Journal, 20*(1), 13-16.

McDermott, C., Richards, S. C., Thomas, P. W., Montgomery, J., & Lewith, G. (2006). A placebo-controlled, double-blind, randomized controlled trial of a natural killer cell stimulant (BioBran MGN-3) in chronic fatigue syndrome. *QJM: An International Journal of Medicine, 99*(7), 461-468. https://doi.org/10.1093/qjmed/hcl063

Meshitsuka, K. (2013). A case of stage IV hepatocellular carcinoma treated by KM900, Biobran, and psychotherapy has presented significant good results. *Personalized Medicine Universe (Japanese Edition), 1*(1), 46-48.

Miura, T., Chiba, M., Miyazaki, Y., Kato, Y., & Maeda, H. (2004/2013). Chemical structure of the component involved in immunoregulation. In *BioBran/MGN-3 (Rice Bran Arabinoxylan Coumpound): Basic and clinical application to integrative medicine* (2nd ed., pp. 14-22). BioBran Research Foundation. (Reprinted from a report of 2004 Annual Meeting of the Japanese society of Applied Glycoscience.)

Noaman, E., Badr El-Din, N. K., Bibars, M. A., Abou Mossallam, A. A., & Ghoneum, M. (2008). Antioxidant potential by arabinoxylan rice bran, MGN-3/biobran, represents a mechanism for its oncostatic effect against murine solid Ehrlich carcinoma. *Cancer Letters, 268*(2), 348-359. https://doi.org/10.1016/j.canlet.2008.04.012

Ohara, I., Onai, K., & Maeda, H. (2002). Modified rice bran improves glucose tolerance in NIDDM adult rats given streptozotocin as neonates. *Aichi Gakusen University Research Studies, 37*, 17-23.

Ohara, I., Tabuchi, R., & Onai, K. (2000). Effects of modified rice bran on serum lipids and taste preference in streptozotocin-induced diabetic rats. *Nutrition research, 20*(1), 59-68. https://doi.org/10.1016/S0271-5317(99)00138-4

Okamura, Y. (2004). The clinical significance of Biobran in the immunotherapy for cancer. *Clinical Pharmacology and Therapy, 14*(3), 289-294.

Ooi, S. L., Pak, S. C., Micalos, P. S., Schupfer, E., Zielinski, R., Jeffries, T., Harris, G., Golombick, T., & McKinnon, D. (2020). Rice bran arabinoxylan compound and quality of life of cancer patients (RBAC-QoL): Study protocol for a randomized pilot feasibility trial. *Contemporary Clinical Trials Communications, 19*, 100580. https://doi.org/10.1016/j.conctc.2020.100580

Pérez-Martínez, A., Valentín, J., Fernández, L., Hernández-Jiménez, E., López-Collazo, E., Zerbes, P., Schwörer, E., Nuñéz, F., Martín, I. G., Sallis, H., Díaz, M. Á., Handgretinger, R., & Pfeiffer, M. M. (2015). Arabinoxylan rice bran (MGN-3/Biobran) enhances natural killer cell-mediated cytotoxicity against neuroblastoma in vitro and in vivo. *Cytotherapy, 17*(5), 601-612. https://doi.org/10.1016/j.jcyt.2014.11.001

Pescatore, F. M., Přestáno, C., & Kichikova, M. (2022). RBAC and its role with the immune system. *Alternative Therapies in Health and Medicine, 28*(1), 8-10.

Petrovics, G., Szigeti, G., Hamvas, S., Mate, A., Betlehem, J., & Hegyi, G. (2016). Controlled pilot study for cancer patients suffering from chronic fatigue syndrome due to chemotherapy treated with BioBran (MGN-3Arabinoxylane) and targeted radiofrequency heat therapy. *European Journal of Integrative Medicine, 8*, 29-35. https://doi.org/10.1016/j.eujim.2016.10.004

Salama, H., Medhat, E., Shaheen, M., Zekri, A.-R. N., Darwish, T., & Ghoneum, M. (2016). Arabinoxylan rice bran (Biobran) suppresses the viremia level in patients with chronic HCV infection: A randomized trial. *International Journal of Immunopathology and Pharmacology, 29*(4), 647-653. https://doi.org/10.1177/0394632016674954

Sudo, N., Tatoe, K., Koyama, N., Kanna, H., Hirayama, K., & Kubo, C. (2001). The basic study of alabinoxlan compound (MGN-3) on the activation of vital defence *Rinshou to Kenkyu (Clinical and Research), 78*(1), 193-196.

Takahara, K., & Sano, K. (2004). The life prolongation and QOL improvement effect of rice bran arabinoxylan derivative (MGN-3, Biobran) for progressive cancer. *Clinical Pharmacology and Therapy, 14*(3), 267-271.

Tan, D. F. S., & Flores, J. A. S. (2020). The immunomodulating effects of arabinoxylan rice bran (Lentin) on hematologic profile, nutritional status and quality of life among head and neck carcinoma patients undergoing radiation therapy: A double blind randomized control trial. *Radiology Journal, The Official Publication of the Philippine College of Radiology, 12*(February), 11-16.

Tazawa, K., Ichihashi, K., Fujii, T., Omura, K., Anazawa, M., & Maeda, H. (2003). The oral administration of the Hydrolysis Rice Bran (HRB) prevents a common cold syndrome in elderly people based on immunomodulatory function. *Journal of Traditional Medicines, 20*(3), 132-141.

Tazawa, K., Namikawa, H., Oida, N., Itoh, K., Yatsuzuka, M., Koike, J., Masada, M., & Maeda, H. (2000). Scavenging activity of MGN-3 (arabinoxylane from rice bran) with natural killer cell activity on free radicals. *Biotherapy: Official journal of Japanese Society of Biological Response Modifiers, 14*(5), 493-495.

Tsunekawa, H. (2004). Effect of long-term administration of immunomodulatory food on cancer patients completing conventional treatments. *Clinical Pharmacology and Therapy, 14*(3), 295-302.

University Hospital Medical Information Network Center. (2010). *Radioprotective effects of the Hydrolyzed Rice Bran (HRB) on acute gastroenteritis for cervical cancer treated with chemoradiation*. University hospital Medical Information Network (UMIN) Center. https://center6.umin.ac.jp/cgi-open-bin/ctr_e/ctr_view.cgi?recptno=R000005179

University Hospital Medical Information Network Center. (2018). *A single center, prospective and randomized controlled study of the efficacy and safety of MGN-3/BioBran for patients with B-cell non-Hodgkin's lymphoma after CR*. University hospital Medical Information Network (UMIN) Center. https://center6.umin.ac.jp/cgi-open-bin/ctr_e/ctr_view.cgi?recptno=R000039342

Yamada, T., Daizo, A., Boindogurung, K., Egashira, Y., Maeda, H., & Sanada, H. (2002). Effects of enzyme-treated rice bran hemicellulose (MGN-3) on experimental liver injury in rats [Abstract] *Journal of Japanese Association for Dietary Fiber Research, 6*(2), 107-107. https://doi.org/10.11217/jjdf1997.6.91

Yu, K. W., Shin, K. S., Choi, Y. M., & Suh, H. J. (2004). Macrophage stimulating activity of exo-biopolymer from submerged culture of Lentinus edodes with rice bran. *Journal of Microbiology and Biotechnology, 14*(4), 658-664.

Zhao, Z., Cheng, W., Qu, W., & Wang, K. (2020). Arabinoxylan rice bran (MGN-3/Biobran) alleviates radiation-induced intestinal barrier dysfunction of mice in a mitochondrion-dependent

manner. *Biomedicine & Pharmacotherapy, 124*, 109855. https://doi.org/10.1016/j.biopha.2020.109855

Zheng, S., Sanada, H., Dohi, H., Hirai, S., & Egashira, Y. (2012a). Suppressive effect of modified arabinoxylan from rice bran (MGN-3) on D-galactosamine-induced IL-18 expression and hepatitis in rats. *Bioscience, Biotechnology, and Biochemistry, 76*(5), 942-946. https://doi.org/10.1271/bbb.110968

Zheng, S., Sugita, S., Hirai, S., & Egashira, Y. (2012b). Protective effect of low molecular fraction of MGN-3, a modified arabinoxylan from rice bran, on acute liver injury by inhibition of NF-[kappa]B and JNK/MAPK expression. *International Immunopharmacology, 14*(4), 764-769. https://doi.org/10.1016/j.intimp.2012.10.012

Zhu, X., Okubo, A., Igari, N., Ninomiya, K., & Egashira, Y. (2017). Modified rice bran hemicellulose inhibits vascular endothelial growth factor-induced angiogenesis in vitro via VEGFR2 and its downstream signaling pathways. *Bioscience of Microbiota, Food and Health, 36*(2), 45-53. https://doi.org/10.12938/bmfh.16-016
