# Supplementary material for: Modified rice bran arabinoxylan as a nutraceutical in health and disease—A scoping review with bibliometric analysis
Source: PLoS One. 2023 Aug 31;18(8):e0290314. doi: 10.1371/journal.pone.0290314 (PMC10470915; doi:10.1371/journal.pone.0290314)
Supplement: Attachment — Submitted filename: Response to Reviewers.pdf [file pone.0290314.s008.pdf]

The authors' responses to each point raised by the academic editor and reviewer(s) are as follows:

Reviewer #1

#	Comments:	Response
1	The authors have addressed all the comments I made. I can consequently see that the manuscript has improved significantly in quality.<br>Well done to the authors.<br>______	We appreciate the reviewer's effort in thoroughly inspecting our work and providing valuable input to improve this manuscript. Thank you very much.

Additional Editor Comments:

#	Comments:	Response
1	The article is still lacking the cohesion between results a and discussion and as indicated by one the reviewer regarding the ethical issue those needs to be resolved . I encourage its resubmission as major revisions.	We are puzzled by the comments made by the editor, as the reviewer has clearly stated that all comments have been addressed and the quality of the manuscript has been significantly improved.<br><br>We are unable to make any further changes to the manuscript unless the editor can provide specific details on how the manuscript "is still lacking the cohesion between results and discussion" and what exactly is the "ethical issue that needs to be resolved".<br><br>Thank you.
