# Supplementary material for: Modified rice bran arabinoxylan as a nutraceutical in health and disease—A scoping review with bibliometric analysis
Source: PLoS One. 2023 Aug 31;18(8):e0290314. doi: 10.1371/journal.pone.0290314 (PMC10470915; doi:10.1371/journal.pone.0290314)
Supplement: Attachment — Submitted filename: Reviewer comments_PONE-D-23-12330.docx [file pone.0290314.s006.docx]

**Reviewer comments**

In this work, Ooi et al. conducted a scoping review on the potential health-promoting effects of modified rice bran arabinoxylan as a nutraceutical ingredient in disease management. Kindly, find below my comments for your perusal.

**Abstract**

The authors should indicate the “databases” where the article search was carried out and the date for article search. In addition, could the authors indicate at least two of the countries that recorded the highest number of research on the RBAC? In this statement “Cancer patients reported reduced side effects from chemoradiotherapy and improved quality of life in human studies………….”, was it when the RBAC was combined with the chemoradiotherapy as an adjuvant? How was the RBAC administered? Was the administered before the intervention? This has to be made clear.

**Keyword**: The authors should kindly add “health effects” and “physiological effects”.

**Introduction**

The authors should kindly highlight the global production capacity of rice bran and how its disposal is a nuisance on the environment and thus the need for valorisation into products including RBAC. A brief insight on how the RBAC is produced from the rice bran will be great.

Line 15-19: The authors should state clearly in what form the RBAC is administered. As a supplement? Also, is it only in cancer treatment the RBAC has been used in past research? Other applications in disease management should be highlighted.

Line 16: The authors indicate “Previous reviews…….” Yet they cited just one reference. This is woefully inadequate reference to support the statement.

**Methods**

Under the section “**S1. Supporting Materials for Methods**”, “S1.1 Search strategy example”, I couldn’t see “health effect* OR physiological effect*” as part of the search strategy listed below. The information there only touches on the “RBAC” and not any of the health effects associated with its use. Could the authors clarify that?

The authors should also state that the selected articles and its information should was presented using a PRISMA guideline as suggested by Page et al. “Page, M. J., McKenzie, J. E., Bossuyt, P. M., Boutron, I., Hoffmann, T. C., Mulrow, C. D., ... & Moher, D. (2021). The PRISMA 2020 statement: an updated guideline for reporting systematic reviews. International journal of surgery, 88, 105906.”

**Eligibility**

Line 46: Regarding the statement “…….or the brand/product name…”, do the authors mean that they focused on only commercially produced RBAC? What about RBAC produced on small scale and didn’t have a BRAND name? Why did the authors exclude those studies?

Line 53-54: Why would the authors include articles published as “Conference proceedings” when in most cases, they don’t go through rigorous peer review to ascertain the validity and credibility of the study data?

**Information sources**

Line 74: Why did the authors include unpublished articles? How would you trust the quality of the data?

**Discussion**

Line 471-472: Kindly, provide references to support the statement

Line 486: Kindly, provide references to support those statements

**Conflict of interest statement**

The authors have acknowledged two individuals at the “Acknowledgement” section that works for these commercial entities that are at the forefront of pushing BRAC for consumer purchase. There could be potential conflict of interest there. The authors have to declare that.

**Other general comments**

1. The authors failed to support most of the statements made under the “Discussion” section with references. This makes the “Discussion” become almost like the “Results” and consequently weakens the Discussion of the review. The authors should kindly discuss this in the context of what others have reported and have done.
2. In the “Results” presentation for example, the authors showed that most of the publication output were recorded from the United States of America, followed closely by Japan, South Korea, Egypt etc. However, the authors failed to discuss this trend in the Discussion. Why was this observed? Could it be attributed to the fact that USA is a leading rice producer and thus generates huge amounts of the rice bran? These have to be highlighted.
3. Even though the authors highlighted the potential health-promoting effects of the BRAC, they failed to indicate the potential mechanism of action and which potential individual compounds in the BRAC that could be driving those physiological effects with the beneficial health-promoting effects observed consequently.
4. The authors should also have assessed the quality of articles that were selected for this review.
5. In fact, if you peruse the total number of references at the Bibliographic references, only 29 references have been indicated. This is unacceptably too small considering the magnitude of this review. The authors should work on this. I find that the authors have additional references, which have been uploaded as part of the supporting information (S4. Additional tables and figures). That Table should be part of the review as it highlights the summary of the study findings.
6. The authors could strengthen the “Discussion” by systematically discussing each of the “Section titles” under the “Results” section. For example “Institutions” etc. The authors should attribute reasons to the observed trends.
