# Supplementary material for: Modified rice bran arabinoxylan as a nutraceutical in health and disease—A scoping review with bibliometric analysis
Source: PLoS One. 2023 Aug 31;18(8):e0290314. doi: 10.1371/journal.pone.0290314 (PMC10470915; doi:10.1371/journal.pone.0290314)
Supplement: Attachment — Submitted filename: Hirut A comments.doc [file pone.0290314.s007.doc]

Dear Editor:

I have been asked to review a manuscript entiled ‘ Modified Rice Bran Arabinoxylan as a Nutraceutical in Health and Disease – A Scoping Review with Bibliometric Analysis’. Below is my comment.

Comment:

The effort in this paper is excellent at providing an overview of researches conducted in different countries and published in different languages about the health benefits of Rice Bran Arabinoxylan compound (RBAC). This information is very essential since it gives basic and in fact well-organized information (by identifying clear gaps in the research area) for researchers who want to pursue their research in the field of Nutraceutical research or specifically on RBAC translational research.

Recommendation:

The manuscript is well written and I recommend to be accepted as it is.

Thanks
